# The effect of FSH and activin A on Akt and MAPK1/3 phosphorylation in cultured bovine ovarian cortical strips

**DOI:** 10.1186/s13048-016-0222-2

**Published:** 2016-03-11

**Authors:** Filiz Tepekoy, Gokhan Akkoyunlu

**Affiliations:** Department of Histology and Embryology, Faculty of Medicine, Akdeniz University, 07070 Campus, Antalya, Turkey

**Keywords:** Activin, FSH, Akt, MAPK

## Abstract

**Background:**

rhFSH and rhActA have been used in mammalian ovarian follicle culture systems for activation of follicular growth in vitro and suggested to be responsible for primordial follicle survival through MAPK and Akt pathways. The aim of our study was to determine the effects of rhFSH and rhActA on Akt, pAkt, MAPK1/3 and pMAPK1/3 protein levels in bovine ovarian cortical strips cultured in vitro.

**Methods:**

Ovarian cortical strips from heifers were cultured in the presence of rhFSH (50 ng/mL), rhActA (100 ng/mL) or combination of these factors for 6 days. The strips were embedded in paraffin for histological observations and homogenized for western blot to determine Akt, pAkt, MAPK1/3 and pMAPK1/3 protein levels after the culture. Determination of primordial, primary and secondary follicle proportions at the end of culture as well as comparison of healthy follicle for each developmental stage after the culture was performed to quantify follicle survival and activation.

**Results:**

pAkt protein levels were significantly lower in rhFSH + rhActA group among the other groups, whereas pMAPK1/3 levels were not significantly changed. Follicular activation and survival was measured to be significantly lower in rhFSH + rhActA group. Percentage of healthy primordial follicles was higher in control group whereas healthy secondary follicle proportion was higher in both rhActA and rhFSH groups. rhActA alone had a better impact on follicular activation, since the percentage of the secondary follicles was significantly higher than other treatment groups.

**Conclusions:**

The use of rhActA and rhFSH alone or in the combined form results in differential levels of Akt and MAPK proteins. Both rhActA and rhFSH alone has a remarkable contribution in survival and activation of the follicles in accordance with higher levels of these proteins. Thus, the manipulation of Akt and MAPK pathways with appropriate activators might contribute to proper activation and development of ovarian follicles in vitro.

## Background

Ovarian folliculogenesis includes growth of follicles gradually and development of competent oocytes. Development of primordial follicles to preantral follicles takes place in a gonadotropin independent manner, whereas development of antral follicles to ovulatory follicles comprises the gonadotropin dependent phase of the folliculogenesis. Direct interactions between granulosa cells and oocytes regulate pre-antral follicle development under the control of two oocyte specific members of the transforming growth factor-β (TGF-β) super family, growth differentiation factor 9 (GDF-9) and bone morphogenetic factor 15 (BMP-15) [1]. Further development of selected ovarian follicles proceeds, whereas most follicles undergo atresia by follicle cell apoptosis [2]. Follicles selected for further development are thought to receive precise signals from gonadotropins and locally produced growth factors for survival, whereas follicular atresia or granulosa cell apoptosis minimally evident [3, 4]. Kit ligand is one of the growth factors that has important roles in maintaining the primordial follicle reserve. In primordial follicles, Kit ligand is expressed by developing granulosa cells and its corresponding receptor is localized to the oocyte membrane [5]. Follicular survival and activation is promoted by stimulation of intracellular phosphoinositide 3-kinase (PI3K)/Akt signaling pathway by Kit ligand in primordial oocytes [6, 7]. The PI3K/Akt signaling pathway is reported to have roles in primordial follicle activation as well as stem cell maintenance and organogenesis [8].

Akt, also known as protein kinase B (PKB), is a serine/threonine protein kinase and its activity is modulated downstream of PI3K in response to many growth factors and cytokines. Akt is recruited to plasma membrane after binding to the 3-phosphoinositide produced by PI3K [9]. Akt isoform Akt1 has been reported to be critical for female fertility. Akt1 is localized in granulosa cells and oocytes of both human [10] and rodent [11] ovaries. In the porcine ovary, Akt1 is localized in granulosa cells of primordial follicles and in the basal layers of the granulosa cells of preantral and antral follicles.

Mitogen activated protein kinases (MAPK) are present in a number of cell types and their roles in cell cycle, proliferation and differentiation are controlled by a series of extracellular signals [12]. MAPK3 (p44ERK1) and MAPK1 (p42ERK2) isoforms of MAPK are found in mammalian oocytes and these kinases are shown to be activated during meiotic maturation [13]. MAPK has been shown to be upregulated by epidermal growth factor/epidermal growth factor receptor (EGF/EGFR) to induce growth of primordial to secondary follicles [14].

Activin is one of the factors responsible for follicle activation and preantral development [15, 16]. Activin was first discovered as a heterodimeric protein composed of the two -β subunits of inhibins A and B linked by interchain disulphide bond(s) and defined as a “FSH-releasing substance”. Since structural organization of Activin was found to be homologous to that of TGF-β, it was included in this superfamily [17]. Three different isoforms of activin referred to as activin A (βA-βA), activin AB (βA-βB) and activin B (βB-βB) are formed by dimerization of –β subunits [18]. Activin has putative biological actions in a wide variety of tissues such as the pituitary, bone, gonad, liver and kidney as well as hematopoietic cells [19, 20].

Though in the former studies, activin was found to be expressed only in the granulosa cells rather than oocytes [21], presence of activin in both oocytes and granulosa cells was proved in rodent [22], porcine [23] and bovine follicles [24]. Activin A was localized in the outer ooplasm and zona pellucida of immature bovine oocytes, whereas it was localized in the zona pellucida, perivitellin space and oolemma after maturation and fertilization [25]. Both types of activin receptors (type 1 and type 2), are expressed in mammalian ovaries [26]. Activin receptors are expressed both in cumulus cells and oocytes [27, 28]. Activin promotes the release of FSH from the anterior pituitary [29]. Additionally, activin has a role in promoting aromatase activity, antral cavity formation, and granulosa cell proliferation [15, 22, 30]. Activin is found to promote FSH receptor expression on undifferentiated rat granulosa cells [31]. Activin A (ActA) is thought to be involved in the early follicular phase FSH rise [32]. Activin function is antagonized by follistatin and inhibin binding to its receptors [33].

Though FSH receptors are present on the granulosa cells of early preantral follicles [34], activation of follicle growth and initial development is accepted to be independent of FSH [35]. Follicle growth was found to become critically dependent on FSH at the emerging antral stage [36]. Besides its roles in antral follicle development, FSH is considered to be related with the actions of activin regarding early follicular development [37], since FSH is found to improve the actions of activin [15, 38]. It has been found that FSH is required for mouse, human and rhesus in vitro pre-antral follicle development [39–41]. In vitro studies on preantral follicle development revealed that ActA decreases the proportion of atretic follicles, whereas combined treatment of ActA with FSH increases the proportion of atretic preantral oocytes [42]. Although, there is evidence that both FSH and ActA are critical for preantral follicle development and survival, their effects on the downstream signaling molecules have not been fully identified in the developing follicles. Hence, in our study we aim to find out the effects of FSH and ActA on p-MAPK and p-Akt protein levels of in vitro cultured bovine ovarian cortical strips including follicles at earlier stages of development.

## Methods

### Isolation and culture of bovine cortical strips

Bovine cortical strips were isolated and cultured as described previously [43]. Briefly, ovaries from heifers were transported at 33–38 °C in HEPES buffered M199 media (Lonza Inc, Walkersville, MD, USA) supplemented with amphotericin B (2.5 mg/ml; Invitrogen Ltd, Paisley, UK), pyruvic acid (25 mg/ml), penicillin G (75 mg/ml) and streptomycin (50 mg/ml; all Sigma Chemicals, Poole, UK) after slaughter in local abattoir (ANET Inc., Antalya, TURKEY). The ovaries were rinsed in 70 % alcohol and fine strips of cortex removed using a scalpel under laminar flow conditions in dissection medium [Leibovitz medium (Lonza Inc, Walkersville, MD, USA) supplemented with sodium pyruvate (2 mM), glutamine (2 mM; both Invitrogen Ltd., Paisley, UK), bovine serum albumin (BSA; Fraction V, 3 mg/ml), penicillin G (75 mg/ml) and streptomycin (50 mg/ml; all Sigma Chemicals, Poole, UK)]. Then, the strips were placed in the culture medium [McCoy’s 5a medium (Lonza Inc, Walkersville, MD, USA) with bicarbonate supplemented with HEPES (20 mM), glutamine (3 mM; both Invitrogen Ltd., Paisley, UK), BSA (Fraction V 0.1 %), penicillin G (0.1 mg/ml), streptomycin (0.1 mg/ml), transferrin (2.5 mg/ml), selenium (4 ng/ml), insulin (10 ng/ml) and ascorbic acid (50 mg/ml), all Sigma Chemicals, Poole, UK] in the 24 well plate. Each well included one cortical strip. Five experimental groups of cortical strips existed: Cortical strips obtained from fresh bovine ovaries (1), cortical strips cultured in vitro without any activation (2) or with activation of rhFSH (50 ng/mL) (R&D Systems, Abingdon, UK) (3), rhActA (100 ng/mL) (R&D Systems, Abingdon, UK) (4) or combination of these activators (5). Each group contained the pool of cortical strips from five different animals and all of the experiments were repeated three times. Culture period was 6 days for all cultured groups at 37 °C in humidified air with 5 % CO_2_ with medium changed every 2 days. The imaging of each strip was performed both in the beginning and at the end of the culture under stereo microscope (Zeiss Stemi SV 11, Oberkochen, Germany). The experimental protocol was approved by the Animal Ethics Committee of Akdeniz University, Turkey (2012.08.29).

### Histological observations

At the end of the culture period cortical strips were fixed by immersion in Bouin’s fixative (75 mL of saturated aqueous solution of picric acid [Sigma-Aldrich Co. LLC, Steinheim, Germany], 25 mL of formalin [Merck, NJ, USA] and 5 mL of glacial acetic acid [Sigma-Aldrich Co. LLC, Steinheim, Germany]) at room temperature for 4 h. Then tissues were dehydrated through a graded series of ethanol, cleared with xylene and finally embedded in paraffin wax. The samples were sectioned (5 μm) and mounted on charged slides then allowed to dry overnight at 37 °C and stained with haematoxylin and eosin for histological observations. Follicles at primordial, primary and secondary stages were counted at the end of the culture and percentage of the follicles of each stage among the total number of follicles were determined for each group and presented as percentage (%). Percentage of the healthy follicles at each stage was also identified after the culture period for each group. Follicles were counted and classified when the oocyte nucleolus was present to avoid double counting. The follicles with an intact oocyte in contact with a complete layer of granulosa cells were considered as healthy follicles. All histological evaluations were performed under the light microscope (Zeiss Axioplan, Oberkochen, Germany).

### SDS polyacrylamide gel electrophoresis and western blotting

In order to perform protein extraction and immunoblot analysis of Akt, pAkt, MAPK1, MAPK3, pMAPK1 and pMAPK3; cortical strips were weighed and put into homogenization buffer (10 mM Tris–HCL, 1 mM EDTA, 2.5 % SDS, 1 mM phenylmethylsulfonylfluoride, 1 μg/mL leu-peptin) supplemented with CompleteR protease inhibitor cocktail (Boehringer, Mannheim, Germany). After homogenization, samples were centrifuged at 10,000 × g for 10 min. Supernatants were collected and stored at −80 °C. The protein concentration was determined by Lowry assay [44] and 50 μg protein was applied per lane. Prior to electrophoresis, samples were boiled for 5 min at 95 °C. Samples were subjected to SDS polyacrylamide gel electrophoresis and then were transferred onto nitrocellulose membranes (Amersham Pharmacia, Piscat-away, NJ, USA) in a buffer containing 0.2 mol/l glycine, 25 mMTris and 20 % methanol overnight. Successful transfer was confirmed by Ponceau S (Sigma-Aldrich Co. LLC, Steinheim, Germany) staining of the blots. The membranes were blocked for 1 h with 5 % non-fat dry milk (BioRad, Hercules, CA, USA) and 0.1 % Tween 20 (Sigma-Aldrich Co. LLC, Steinheim, Germany) in 0.14 mol/l Trisbuffered saline (TBS) pH:7.2–7.4 at 4 °C. Blotting membranes were incubated overnight at 4 °C with Akt, pAkt, MAPK1/MAPK3 and pMAPK1/pMAPK3 (Cell Signaling Tech., Danvers, MA, USA) antibodies at 1:1000 dilution. After washing steps, the membranes were further incubated with goat anti rabbit IgG horseradish peroxidase conjugate (BioRad, Her-cules, CA, USA) diluted 1:5000 for 1 h at room temperature. Immunolabeling was visualized using the chemiluminescence based SuperSignal CL HRP Substrate System (Pierce, Rockford, IL, USA) and the membranes were exposed to Hyperfilm (AmershamPharmacia). β-Actin antibody (1:5000 dilution) (Abcam, Cambridge, UK) was used as an internal control for each blotting in order to confirm the equal loading of the samples. The bands were quantified using NIH image analysis software (ImageJ Version 1.36b, National Institutes of Health, Bethesda, MD, USA).

### Statistical analysis

The data obtained from ImageJ for western blotting experiment were analyzed with non-parametric ANOVA on ranks (Kruskal–Wallis test) and parametric One-way ANOVA, Holm Sidak method. The values were presented as mean ± SEM. Statistical calculations were performed using Sigma Stat for Windows, version 3.0 (Jandel Scientific Corp. San Rafael, CA, USA). Statistical significance was defined as *P* <0.05.

## Results

### Assessment of the follicle health and activation

**Fig. 1:**
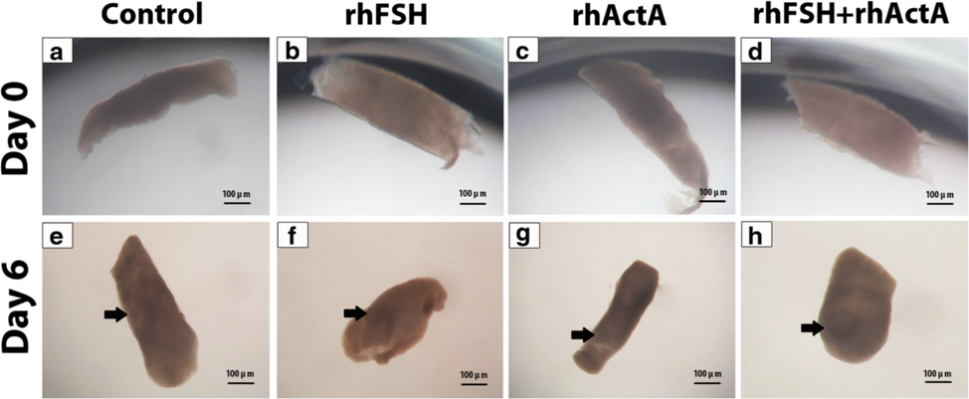
Stereomicroscopic images of cortical strips. Control (**a**), rhFSH (**b**), rhActA (**c**), rhFSH + rhActA (**d**) groups in the beginning of the culture (The cover of the plate was closed during photographing to preserve aseptic conditions). Control (**e**), rhFSH (**f**), rhActA (**g**), rhFSH + rhActA (**h**) groups at the end of the culture. Arrow: Secondary follicles

**Fig. 2:**
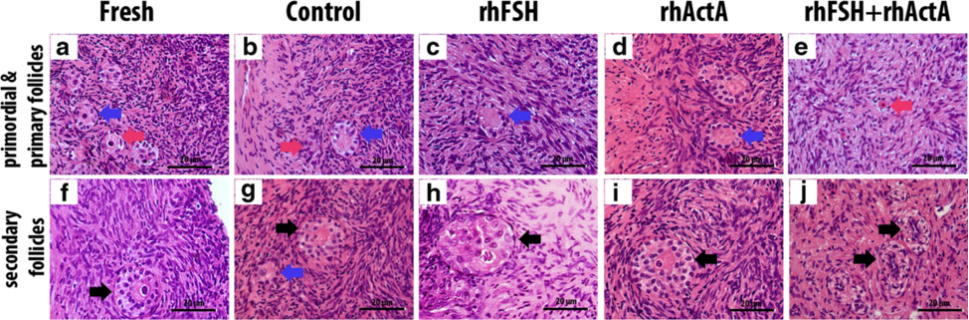
Haematoxylin and eosin staining of fresh and cultured cortical strips. Section from uncultured fresh (**a**, **f**), control group of day 6 of culture (**b**, **g**), day 6 of culture supplemented with rhFSH (**c**, **h**), rhActA (**d**, **i**) and rhFSH + rhActA (**e**, **j**). Red arrows: Primordial follicles. Blue arrows: Primary follicles. Black arrows: Secondary follicles

Bovine cortical strips isolated (Fig. 1a, b, c, d) cultured in vitro were observed to harbor secondary follicles at the end of the culture (Fig. 1e, f, g, h). When percentage of primordial, primary and secondary follicles were evaluated at the end of 6 days of culture via histological observations (Fig. 2), it was found that rhActA group had significantly highest and rhFSH + rhActA group had significantly lowest percentage of secondary follicles. 7 % of follicles from control group were secondary follicles. There were 12 and 20 % of secondary follicles in rhFSH and rhActA treated groups respectively, whereas the proportion of secondary follicles was 5 % in combination group of rhFSH + rhActA. The significantly higher percentage of secondary follicles in rhActA group pointed out the remarkable activating effect of rhActA on the follicles (Table 1). At the end of the 6 days of culture period, rhFSH + rhActA group cortical strips were observed to have the most impaired morphology (Fig. 2e, j), whereas control (Fig. 2b, g), rhFSH (Fig. 2c, h) and rhActA (Fig. 2d, i) groups have morphological patterns closer to uncultured fresh cortical strips (Fig. 2a, f). After identification of percentage of healthy follicles at different stages after the culture period, it was found that rhActA group as well as rhFSH group had significantly higher percentage of healthy secondary follicles than the other groups. In terms of primordial follicles, control group had significantly the highest percentage among other groups. rhActA + rhFSH group showed the lowest percentage of healthy primordial and secondary follicles at the end of the culture. rhActA and rhFSH groups had higher percentage of primary follicles when compared to the other groups though this difference was not statistically significant (Table 2).

**Table 1:** Percentage of follicles at different stages at the end of 6 days of culture

	Control	rhFSH	rhActA	rhFSH + rhActA
Primordial	78.7 ± 1.1^a^	70.4 ± 1.7^b^	57.6 ± 2.8^c^	81.8 ± 2.2^a^
Primary	13.8 ± 0.7^a^	17.7 ± 3.6^b^	22.0 ± 1.0^c^	13.2 ± 1.5^a^
Secondary	7.4 ± 0.5^ab^	11.5 ± 1.6^b^	20.2 ± 2.6^c^	4.9 ± 1.0^a^

Among columns: a, b, c (*P* < 0.05)

**Table 2:** Percentage of healthy follicles at the end of 6 days of culture

	Control	rhFSH	rhActA	rhFSH + rhActA
Primordial	65.4 ± 2.3^a^	55.4 ± 3.0^ab^	60.2 ± 3.1^ab^	52.3 ± 1.9^b^
Primary	60.0 ± 2.0^a^	65.0 ± 2.5^a^	68.1 ± 2.1^a^	60.0 ± 2.7^a^
Secondary	62.4 ± 2.0^a^	70.3 ± 2.2^b^	73.0 ± 1.3^b^	51.4 ± 2.9^c^

Among columns: a, b, c (*P* < 0.05)

### Phosphorylated Akt protein levels in cultured bovine cortical strips

**Fig. 3:**
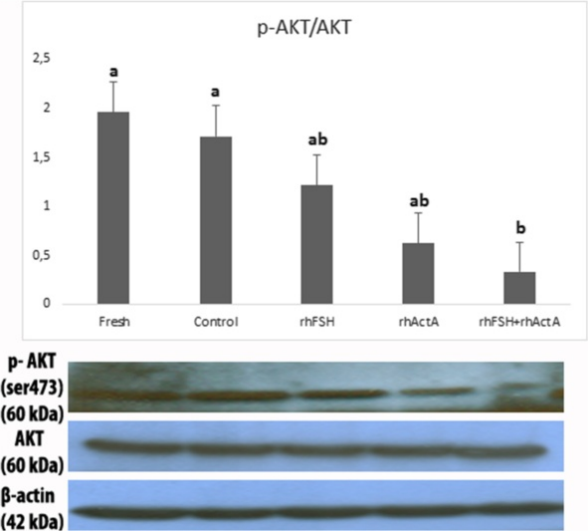
Western blot bands and graphics of mathematical values of ImageJ evaluations of pAkt/Akt protein levels in postnatal mouse ovaries. Different letters mark statistical significance (*P* < 0.05) (One way anova, Holm Sidak method)

Phosphorylated Akt protein level of fresh bovine cortical strips was higher when compared to the culture groups. pAkt protein level of control group cortical strips was closer to fresh cortical strips among other culture groups. pAkt levels was higher in rhFSH group when compared to the rhActA and rhFSH + rhActA culture groups. rhFSH + rhActA group displayed significantly (*P*˂0.05) the lowest level of pAkt when compared to fresh and control groups (Fig. 3).

### Phosphorylated MAPK protein levels in cultured bovine cortical strips

**Fig. 4:**
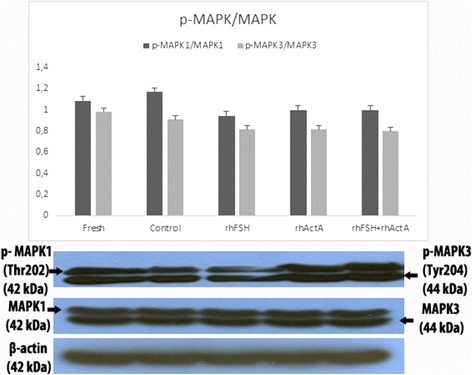
Western blot bands and graphics of mathematical values of ImageJ evaluations of pMAPK1/3 / MAPK1/3 protein levels in postnatal mouse ovaries. Different letters mark statistical significance (*P* < 0.05) (One way anova, Holm Sidak method)

Phosphorylated MAPK1 and MAPK3 protein levels of fresh and control bovine cortical strips were also higher when compared to the other culture groups though this difference was not statistically significant. There was a slight decrease in p-MAPK1 level of rhFSH group when compared to the other groups and this difference was not statistically significant either (Fig. 4).

## Discussion

Findings of the current study indicate that involvement of rhFSH and rhActA in cortical strip culture of bovine ovaries significantly affects pAkt levels that have remarkable roles during early folliculogenesis. When rhActA was applied in combination with rhFSH, pAkt protein displayed significantly the lowest level. Akt is known to be phosphorylated at two sites: the Thr308 residue phosphorylated by the phosphoinositide-dependent kinase 1 (PDK1) [45]; the Ser473 residue phosphorylated by the mammalian target of rapamycin (mTOR) [46] as well as integrin-linked kinase (ILK) [47] and mitogen-activated protein kinase-activated protein kinase-2 (MAPKAPK2) [48]. Since we investigated the levels of pAkt phosphorylated at Ser473, the decrease in the pAkt levels in the presence of rhActA and rhFSH might be associated with impairment of mTOR, ILK or MAPKAPK2.

In previous studies, the effects of rhFSH and rhActA on ovarian follicles cultured in vitro were assessed in different species in a morphological manner. rhFSH and rhActA, both alone and in the combined form was included in human cortical strip culture [49] or bovine [37, 50], human [43] and primate [51] pre-antral follicle culture followed by strip culture as well as rodent ovary organ cultures [42, 52] and their effects on in vitro follicle development was evaluated. Proportion of human pre-antral follicles cultured in vitro reaching antral stage was reported to be higher in the presence of rhActA [43], whereas another study showed that rhActA had a better impact on bovine ovarian follicle diameter when used in the combined form with rhFSH, though oocyte diameter alone was not affected by these combination [50]. It was also reported that rhActA alone had a better impact on oocyte morphology of cultured bovine preantral follicles when compared to rhFSH or combination of rhActA and rhFSH groups of culture [37]. On the other hand, rhActA was reported to have an inhibitory effect on human primordial follicle activation [49]. Reversely, in our study conducting bovine ovarian strip culture, we achieved an enhancement in secondary follicle development when we applied rhActA alone. rhFSH was reported to promote nest breakdown and primordial follicle formation at low levels of estradiol (E2) in rodents [52], whereas pre-antral follicle culture of primates revealed that rhFSH disrupted the integrity of oocyte and cumulus cells resulting in impaired follicle health [51]. In our study, addition of rhFSH alone did not cause a remarkable disruption in the follicles developed until the secondary stages.

For an effective evaluation of the ovarian follicles developed in vitro, the signaling components affecting the developmental potential of the follicles must also be considered through molecular techniques. There are particular studies suggesting an enhancement in FSH receptor, activin βA, βB subunit m-RNA levels in neonatal [42] and fetal rodent ovary culture model [52] in the presence of rhActA [42] and rhFSH [52]. In the current study, rhActA alone had a better impact on follicular development when compared to rhFSH. However, this impact was not reflected to the enhancement of p-Akt and p-MAPK levels that have critical roles in follicle development and survival [53, 54]. Interestingly, control group of culture which had the highest level of these proteins among the culture groups, also had the highest percentage of healthy primordial follicles. Thus, it can be suggested that, especially p-Akt levels that were significantly affected by culture conditions, might be more critical for primordial follicles rather than further stages.

FSH significantly affects structure and function of follicular cells at later stages of follicular development in association with different signaling pathways. In granulosa and theca cell cultures, FSH actions on hormone production was reported to be Akt and MAPK dependent. Addition of FSH in the granulosa cell culture was shown to enhance phosphorylation levels of Akt and MAPK [55]. FSH induces Akt phosphorylation in granulosa cells resulting in their differentiation [56]. FSH is also known to act on granulosa cell proliferation through the activation of the MAPK pathway [57] besides the Akt pathway [58] and the inositol triphosphate and diacylglycerol pathways [59]. In the current study, though rhFSH alone had a positive effect on follicle development until the secondary stage and secondary follicle survival, the percentage of healthy primordial follicles were low in rhFSH group when compared to the control group. The reason behind these observations might be that p-Akt and p-MAPK1/3 levels were not enhanced in rhFSH group. Though FSH was shown to enhance Akt phosphorylation in cultured granulosa cells [55–58], in our study a significant effect of FSH on Akt phosphorylation was not observed in cortical strip cultures that specifically include ovarian follicles at primordial, primary and secondary stages. When FSH was evaluated in terms of its effects on molecular aspects via ovarian organ culture of immature rats that include only primordial and primary follicles, it was revealed that it had no significant effect on folistatin, activin receptor and FSH receptor mRNAs [42]. Thus, the effect of FSH on different signaling molecules must be investigated through both in mRNA and protein levels.

In previous studies, EGF, as an upstream regulator of MAPK and PI3K signaling pathways, was shown to play a significant role in maintaining intraovarian primordial follicle viability and promoting ovarian cell proliferation in the prepubertal cat [54]. There is evidence that during folliculogenesis granulosa and cumulus cells become responsive to TGF-β superfamily growth factors. EGF receptor– MAPK1/3 pathway was shown to be enabled by GDF9–SMAD3 signaling in granulosa and cumulus cells [60]. Physiological concentrations of LH and FSH was shown to increase enzymatic activity of MAPK3 but not that of MAPK1 in the cytosol and of both MAPK1 and MAPK3 in the nucleus of porcine granulosa cells. Activation of MAPK3 by gonadotropins as well as cAMP was accompanied by increased tyrosine phosphorylation of the kinase. Following treatment with gonadotropins, translocation of MAPK to the nucleus was shown in porcine granulosa cells. EGF was reported to increase MAPK1 and MAPK3 associated kinase activity 7–8-fold in the cytoplasm of porcine granulosa cells, while kinase activity of cytoplasmic MAPK3 was enhanced 3–4-fold by LH, FSH, or cAMP [61]. In the current study, phosphorylated MAPK1 and MAPK3 levels were not significantly affected by addition of rhFSH or rhActA in the culture. In order to obtain an enhancement in the phosphorylation and activation of these proteins to maintain the follicular development, addition of growth factors such as EGF that has a direct effect on these proteins might be essential.

Primordial follicle activation was closely associated with PI3K/Akt pathway in recent studies conducted in both human [62] and mouse [63]. It was reported that, when phosphatase and tensin homolog (PTEN), a suppressor of PI3K signaling, was inhibited, follicles were induced to progress to the secondary stage and increased activation of follicles was associated with increased Akt phosphorylation and nuclear export of FOXO3 (forkhead family transcription factor), though this application resulted in alterations in the survival of isolated secondary follicles [62]. In a transgenic mice model, in which PI3K from oocyte was constitutively active, apoptosis rate was significantly reduced which resulted in an excess number of follicles per ovary. On the other hand, PTEN was introduced as a preventer of immature follicle activation, since neonatal Cre + mice was shown to remain dormant demonstrating a nuclear accumulation of PTEN. Akt phosphorylation was also increased in these follicles [63]. Thus in our study, rhActA + rhFSH group having the lowest percentage of secondary follicles and the most impaired morphology at the end of the culture might be linked to the significantly low levels of p-Akt in this group of culture.

Though our study was limited to the treatment of single concentrations of follicle activators, according to our current results it can be suggested that use of these activators alone rather than in the combined form has a better impact on early follicular development. Different concentrations of these activators might also have various effects on the morphology of the follicles even eliminating the detrimental effect of rhFSH and rhActA in the combined form which was determined for preantral follicles in previous studies [42]. In order to better understand the effects of these variable treatments, the follicles must be assessed in terms of the protein levels associated with follicular activation and survival besides their morphology.

## Conclusion

In conclusion, this study reveals that treatment of the bovine ovarian cortical strips including follicles at earlier stages of development with combination of rhFSH and rhActA results in impairment of Akt phosphorylation that has key roles in follicular development.

During follicle culture, activators of folliculogenesis might have variable effects on different signaling components. These effects might be long-lasting and sustained until the later stages of follicle development and might even cover the oocyte maturation process. In order to take the advantage of controlling in vitro follicle development considering the key signaling pathways critical for follicular activation and survival at each follicular stage, further studies detecting the levels of a wide range of proteins must be conducted. Thus, controlling the levels of specific signaling members in the follicle culture can lead to retrieve oocytes with an enhanced developmental potential.
